# LINC01232 exerts oncogenic activities in pancreatic adenocarcinoma via regulation of TM9SF2

**DOI:** 10.1038/s41419-019-1896-3

**Published:** 2019-09-20

**Authors:** Qian Li, Chengbin Lei, Changliang Lu, Jingye Wang, Min Gao, Wei Gao

**Affiliations:** 1 Department of Laboratory Medicine, Weifang Medical University, No. 7166, Baotong West Street, Weifang, 261053 China; 2 Department of Clinical Laboratory, Central Hospital of Zibo, NO. 54, Gong Qing Tuan Xi Road, Zibo, 255036 China; 3 School of Clinical Medicine, Weifang Medical University, No. 7166, Baotong West Street, Weifang, 261053 China; 4 Department of Pathology, Weifang Maternal and Child Health Care Hospital, Weifang, 261011 China; 5 Department of Otolaryngology, Weifang People’s hospital, Weifang, Shandong 261000 China; 6 Key Lab for Immunology in Universities of Shandong Province, Weifang Medical University, No.7166, Baotong West Street, Weifang, 261053 China

**Keywords:** Cancer, Cell biology

## Abstract

Pancreatic adenocarcinoma (PAAD), one of the most prevailing malignant tumors in digestive system, is identified as one of the main culprits of cancer-associated mortality. Despite long intergenic non-protein coding RNA 1232 (LINC01232) is found to be upregulated in TCGA PAAD tissues and associated with poor prognosis, the potential of LINC01232 in PAAD progression still needs more explorations. In this study, LINC01232 was chosen to be the research object in PAAD cellular processes. Functionally, loss-of function assays were carried out and the experimental results indicated that suppression of LINC01232 hindered the deterioration of PAAD by affecting cell proliferation and migration. Furthermore, relationship between LINC01232 and its nearby gene transmembrane 9 superfamily member 2 (TM9SF2) was investigated. The same expression pattern of TM9SF2 in TCGA PAAD samples was observed. It was found that upregulation of LINC01232 could be a crucial factor for the dysregulation of TM9SF2. Mechanistically, LINC01232 recruited EIF4A3 to boost TM9SF2 mRNA stability. Besides, our findings demonstrated that the transcriptional activation of LINC01232 and TM9SF2 was mediated by SP1. Therefore, we concluded that LINC01232 executed carcinogenic properties in PAAD progression via regulation of TM9SF2. In conclusion, this study was the first to unveil the role and molecular mechanism of LINC01232, suggesting LINC01232 as a promising molecular target for pancreatic cancer treatment.

## Introduction

Pancreatic adenocarcinoma (PAAD), one of the most prevailing malignant tumors in digestive system, is defined as one of the main culprits of cancer-associated mortality, which constitutes 1–2% of generalized malignancies, which results in more than 227,000 deaths each year worldwide^1^. Despite surgery is one of the appropriate therapeutic regimens for patients with pancreatic adenocarcinoma, but only 10–20% of patients can be surgically resected in view of lacking main signs or symptoms in the early stages and its aggressive malignant behavior^2^. Despite great efforts dedicated in the development of PAAD treatment, the prognosis of patients is far from satisfactory and the 5-year survival rate remains less than 5%^3,4^. Therefore, in-depth investigations on the potential molecular mechanism in PAAD progression and identification of the functional therapeutic targets for the therapy of PAAD are urgently needed.

Long noncoding RNAs (lncRNAs) are a kind of RNA molecules longer than 200 nucleotides without protein-coding potential^5^. Accumulating evidence has exposed that lncRNAs are involved in the initiation and development of numerous malignancies, including pancreatic adenocarcinoma^6,7^. For example, long noncoding RNA CASC2 upregulates PTEN to suppress pancreatic adenocarcinoma metastasis by downregulating miR-21^8^. LncRNA SLCO4A1-AS1 promotes growth and invasion of bladder cancer through sponging miR-335-5p to upregulate OCT4^9^. LncRNA LINC00460 promoted colorectal cancer cells metastasis via sponging miR-939-5p^10^. Through using TCGA data, we determined that long intergenic nonprotein-coding RNA 1232 (LINC01232) is an lncRNA that is upregulated in PAAD samples and predicted poor prognosis. Thus, we chose LINC01232 for functional and mechanism analysis in PAAD cell lines.

LncRNAs are crucial regulators for their nearby genes in human cancers^11–13^. In our current study, we identified that LINC01232 positively regulated its nearby gene transmembrane 9 superfamily member 2 (TM9SF2). A recent study has indicated that TM9SF2 acts as a novel oncogene in colorectal cancer^14^. Nevertheless, the participation of TM9SF2 in pancreatic carcinoma progression is vague. Through bioinformatics analysis and mechanism investigations, the positive regulatory effect of LINC01232 on TM9SF2 was determined in PAAD cell lines. Functionally, the oncogenic potential of LINC01232 and TM9SF2 was determined in PAAD cells. In vitro and in vivo rescue assays demonstrated the interaction between LINC01232 and TM9SF2 in PAAD tumorigenesis. Taken together, our present study revealed that a novel molecular pathway in PAAD cellular activities, thus providing potential therapeutic targets in PAAD treatment.

## Materials and methods

### Clinical samples

Totally, 40 pairs of samples used in current study were collected from patients with PAAD who were diagnosed at Central Hospital of Zibo. Patients participated in this study were not treated with chemotherapy or radiotherapy. Ethical approval has been acquired from the ethics committee of Central Hospital of Zibo. Informed consents were signed by all participants before they enrolled in this study.

### Cell culture

All cell lines used in this study, including human PAAD cell lines (CAPAN-1, BxPC-3, JF305, PANC-1, and SW1990) and human normal pancreatic duct epithelial cell line HPDE6-C7 were acquired from the American Type Culture Collection (ATCC, Manassas, VA, USA) and incubated in DMEM (Gibco, Carlsbad, CA, USA) containing 10% fetal bovine serum (FBS) (Invitrogen, Carlsbad, CA, USA) at 37 °C in a humid atmosphere of 5% CO_2_.

### Actinomycin D treatment

To block transcription, cell culture medium was added with 2 mg/ml Actinomycin D (Sigma-Aldrich, St. Louis, MO, USA) after transfection. After treatment with Actinomycin D for different time points, the remaining of mRNA was assessed using quantitative real-time polymerase chain reaction (qRT-PCR).

### Cell transfection

Short hairpin RNA against LINC01232 (sh-LINC01232#1/2/3), EIF4A3 (sh-EIF4A3#1/2), or SP1 (sh-SP1#1/2) and scrambled shRNA (sh-NC) were applied for knockdown of LINC01232, EIF4A3, or SP1. The full length of TM9SF2, EIF4A3, or SP1 was ligated into the pcDNA3.1 vectors (Genepharma, China) to obtain pcDNA3.1/TM9SF2, pcDNA3.1/EIF4A3, or pcDNA3.1/SP1 plasmid with empty vector as a negative control. Cell transfection was conducted by using Lipofectamine 3000 (Invitrogen) based on the manufacturer’s protocol. ShRNA sequences used in this study were listed as follows: sh-NC: CCGCGGACTTGCCTCCTACACTACTCHAGTAGTGTAGGAGGCAAGTCCTTTTTG; sh-LINC01232#1: CCGCGACACGTCATCTAGAATAACTCHAGTTATTCTAGATGACGTGTCTTTTTG; sh-LINC01232#2: CCGCCAAGAGTGCTGGATCTAAACTCHAGTTTAGATCCAGCACTCTTGTTTTTG; sh-LINC01232#3: CCGCGATTGGTTGCTTTCTGCAACTCHAGTTGCAGAAAGCAACCAATCTTTTTG; sh-EIF4A3#1: ACCTCGCTGCTCAAAGAGGAAGACATTCAAGAGATGTCTTCCTCTTTGAGCAGCTT; sh-EIF4A3#2: ACCTCGCAGATCATCAAAGGGAGAGATCAAGAGTCTCTCCCTTTGATGATCTGCTT; sh-SP1#1: ACCTCGAGGAAGTGGAGGCAACATCATCAAGAGTGATGTTGCCTCCACTTCCTCTT; sh-SP1#2: ACCTCGGGAACATCACCTTGCTACCTTCAAGAGAGGTAGCAAGGTGATGTTCCCTT; sh-TM9SF2#1: ACCTCGCATTATGAATTCCCTGGTCATCAAGAGTGACCAGGGAATTCATAATGCTT; sh-TM9SF2#2: ACCTCGCTATGTTGCTGCCAGATTCTTCAAGAGAGAATCTGGCAGCAACATAGCTT

### Quantitative real-time polymerase chain reaction

Total RNA from PANC-1 and SW1990 cells was isolated with Trizol (Invitrogen) and reverse transcription was conducted with a Reverse Transcription kit (Thermo Fisher Scientific, Waltham, MA, USA). Then, cDNA was subjected to PCR in a Thermal Cycler Dice Real Time system (Takara, Tokyo, Japan) using SYBR Green PCR master mix (Takara). Experiments were repeated three times and the 2^−ΔΔCt^ method was utilized for quantitation of gene expression. GAPDH was employed as normalization controls and primers used for qRT-PCR were listed as follow: LINC01232: 5′-AGGATGCGCCTAAGAAAGGG-3′ (F) and 5′-CCGGGGGATTGAGGAAACAT-3′; EIF4A3: 5′-GGCACAGGAAAAACAGCCACCT-3′ (F) and 5′-TGTAGTCACCGAGAGCAAGCAG′ (R); SP1: 5′-GTCATACTGTGGGAAACG′ (F) and 5′-GCAAATTTCTTCTCACCTGTG′ (R); TM9SF2: 5′-CCTCCAAGAAAAGGGATGCTGC′ (F) and 5′-ACAGGACCACAGCACACGTCAT′ (R); GAPDH: 5′-GCATCCTGGGCTACACTG-3′ (F) and 5′-ACTTCAGGAGCATCTGAAATAGGT-3′ (R).

### MTT assay

3-[4,5-dimethylthiazol-2-yl]-3,5-diphenyl tetrazolium bromide (MTT) assay was performed to assess cell viability. Cells were inoculated into a 96-well plate at a density of 2000 cells/well and cultured at 37 °C. 0, 24, 48, 72, or 96 h later, cells were treated with MTT (5 mg/ml) and underwent 2 h of incubation at 37 °C. Afterwards, the media were removed and 200 μl of DMSO was added to dissolve formazan crystals. Absorbance was examined with a microplate reader (BioRad, Hercules, CA, USA) at 570 nm.

### Colony formation assay

For colony formation assay, 1000 cells/well cells were planted and grown in 12-well plates in a humidified atmosphere for 14 days. The medium was changed every 3 days. Thereafter, the colonies were fixed, stained, and finally calculated.

### Transferase-mediated dUTP nick end labeling (TUNEL) staining assay

TUNEL assay was applied for detection of cell apoptosis using a detection kit (Roche, Mannheim, Germany) in line with the manufacturer’s protocol. The nuclei containing fragmented DNA were stained by the TUNEL kit and served as TUNEL-positive cells. DAPI was applied to double-stain the nucleus and apoptotic cells were visualized with a fluorescence microscopy (Olympus) in five randomly selected view fields.

### Caspase-3 activity assay

The caspase-3 activity assay was performed with a caspase-3 activity kit (Beyotime Biotechnology, Jiangsu, China). After transfection, cells were lysed with RIPA buffer and collected by centrifugation. Then, cell lysates were supplemented with 10 μl capsase-3 substrate Ac-DEVD-pNA and incubated for 1 h. The activity of caspase-3 was estimated by examination of absorbance at 405 nm.

### Flow cytometry analysis of apoptosis

Apoptosis was detected by using flow cytometry analysis in accordance with previous study^15^.

### Transwell assays

Cell migration and invasion were determined by transwell assays. Totally, 1 × 10^4^ cells were seeded to the top of transwell plates with 8 µm pores (Corning Costar, Corning, NY, USA) for cell migration assay. For cell invasion assay, the inserts coated with Matrigel (BD Biosciences San Diego, CA, USA) were applied. 10% FBS was added to the underside of the inserts as the chemoattractant. After incubation for 24 h, migrated or invaded cells were fixed with methanol, stained by 1% crystal violet solution and counted under a light microscope in five random view fields.

### Western blot

Total protein from pancreatic cancer cells was obtained utilizing RIPA lysis buffer (Sigma-Aldrich, St. Louis, MO, USA) with protease inhibitor cocktail (Roche). Then, total protein was detached by 10% sodium dodecyl sulphate polyacrylamide gel electrophoresis gel and transferred to polyvinylidene fluoride membrane (Millipore, Billerica, MA, USA). Afterwards, membranes were blocked in 5% defatted milk at room temperature for 1 h, went through overnight incubation with primary antibody against E-cadherin, N-cadherin, Vimentin, Snail, Twist, or GAPDH (Abcam, Cambridge, UK, USA) at 4 °C. After incubating with horseradish peroxidase (HRP)-conjugated secondary antibody at room temperature for 2 h, immunoblots were examined with an enhanced chemiluminescence substrate (Applygen Technologies, Inc. Beijing, China) and quantified by ImageJ software.

### Chromatin immunoprecipitation (ChIP) assay

The EZ ChIP Chromatin Immunoprecipitation Kit (Millipore, Bedford, MA, USA) was used to conduct ChIP assay in accordance with the manufacturer’s instructions. In brief, transfected cells were incubated with formaldehyde for 10 min to form DNA–protein cross-links, quenched using 0.125 M glycine, followed by centrifugation at 4°C for 5 min at 800*g* and then lysed by sodium dodecyl sulfonate lysis buffer with a protease inhibitor cocktail. Cross-linked cell lysates were sonicated and then immunoprecipitated with antibodies against SP1 (Cell Signaling Technology, Danvers, MA, USA). Input was employed as positive control and IgG served as negative control. The protein A-Sepharose magnetic beads were utilized to collect antibody-bound complexes and subsequently immunoprecipitates were eluted by ChIP-elution buffer. The enrichment of LINC01232 and TM9SF2 promoters was analyzed by qRT-PCR assay.

### RNA immunoprecipitation (RIP) assay

RIP experiments were implemented with the Magna RIP RNA-Binding Protein Immunoprecipitation Kit (Millipore Corporation, Burlington, MA, USA). Cell extracts were harvested with RIP lysis buffer and mixed with magnetic beads and antibodies against EIF4A3 (Abcam). Anti-IgG antibody (Abcam) was utilized as control. The co-precipitated RNAs were purified and subjected to qRT-PCR analysis.

### Subcellular fractionation assay

Cytoplasmic and Nuclear RNA Purification Kit (Norgen, Thorold, ON, Canada) were employed to detach RNA in cytoplasm or nucleus. In brief, cells were collected, lysed on ice, followed by centrifugation at 12,000*g* for 3 min. The supernatant was analyzed for the cytoplasmic RNA and the nuclear pellet was used for detection of the nuclear RNA with U6 as nuclear control and GAPDH as cytoplasmic control.

### Fluorescence in situ hybridization (FISH) assay

Cells were fixed with 4% paraformaldehyde and then treated with 0.5% Triton X-100. Hybridization was implemented with DIG-labeled LINC01232 probe in a dark at 37 °C overnight. Subsequently, cells were washed using 2× saline-sodium citrate, incubated with HRP-conjugated anti-DIG secondary antibodies (Jackson, West Grove, PA, USA) overnight at 4 °C and treated with DAPI for nuclear counterstain. Images were obtained with Olympus confocal laser scanning microscope.

### Luciferase report assay

HEK293 cells were plated into 24-well plates and transfected with pcDNA3.1/SP1 plasmid or empty vector pcDNA3.1, luciferase reporter plasmid containing the promoter of LINC01232 or TM9SF2 and pRL-TK vector by Lipofectamine 3000 (Invitrogen) with PRL-TK as the internal control. Forty-eight hour after transfection, luciferase activity was determined by a Dual-Luciferase Reporter Assay System (Promega, Madison, WI, USA) in light of the manufacturer’s instructions.

### RNA pull-down assay

Pierce Magnetic RNA–Protein Pull-down kit (Thermo Fisher Scientific) was used to perform RNA pull-down assay obeying the manufacturer’s instructions. In short, biotin-labeled sense and antisense LINC01232 were obtained with MEGAscript kit (Ambion, Carlsbad, CA) and Pierce RNA 3′ Desthiobiotinylation Kit (Thermo Scientific, Rockford, IL, USA). Subsequently, 50 pmol biotin-labeled LINC01232 was incubated with cell lysates and 50 μL streptavidin magnetic beads with rotation. The eluted proteins were checked by western blot.

### Immunofluorescence staining assay

After transfection, cells were collected, fixed in 4% paraformaldehyde, permeabilized by phosphate-buffered saline supplemented with 0.1% Triton X-100, incubated with primary antibody against E-cadherin or N-cadherin (Cell Signaling Technology) overnight at 4 °C and stained by secondary antibody conjugated with TexasRed or fluorescein isothiocyanate (Vector Laboratories, Inc., Burlingame, CA, USA) for 1 h at room temperature. The nuclei were counterstained with DAPI (Roche) and immunofluorescence images were captured under a fluorescence microscope (Olympus).

### In vivo tumor formation assay

Four-week-old female BALB/C athymic nude mice used for animal experiment were obtained commercially from National Laboratory Animal Center (Beijing, China). All experimental and animal care procedures were approved by the Animal Research Ethics Committee of Central Hospital of Zibo. Totally, 2 × 10^6^ SW1990 cell lines transfected with sh-NC, sh-LINC01232, or sh-LINC01232 + TM9SF2 were collected. The treated cells were subcutaneously injected into the left flank of the nude mice. Tumor volume was calculated as 0.5 × length × width^2^ every fourth day. Four weeks later, mice were killed. Cancerous tissues were excised and weighed for further study.

### Statistical analysis

All statistical analyses were conducted with SPSS 22.0 and all results of experiments were displayed as mean ± SD. Student’s *t* test was adopted for analysis of differences between two groups. Comparisons among three or more groups were analyzed by one-way ANOVA. *P* value threshold was set as 0.05 to be statistically significant.

## Results

### LINC01232 expression is upregulated in PAAD samples and associated with poor patients’ prognosis

**Fig. 1:**
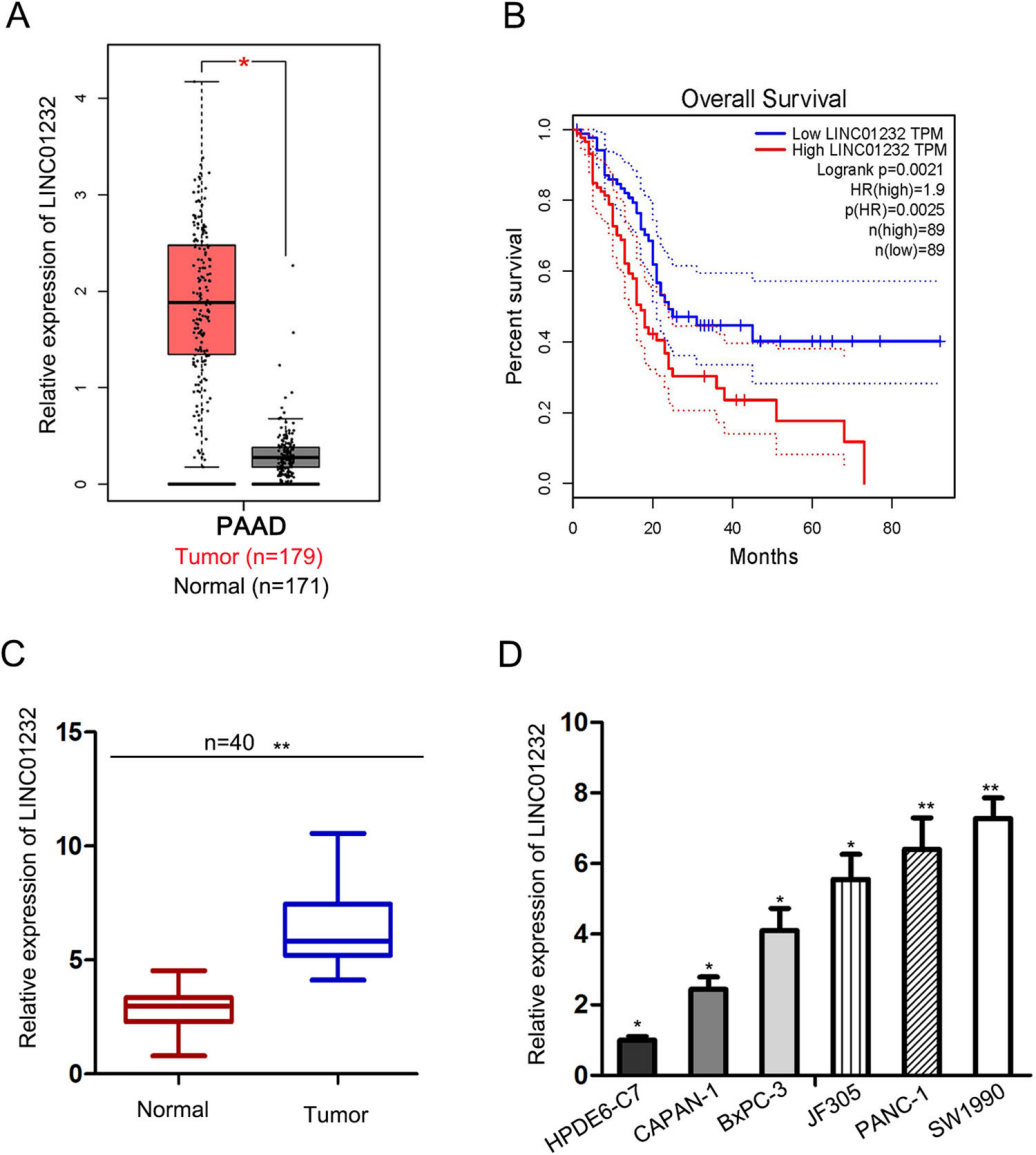
LINC01232 expression is upregulated in PAAD samples and associated with poor patients’ prognosis. **a** TCGA data of LINC01232 expression in PAAD and normal samples. **b** TCGA survival analysis of PAAD patients with high or low LINC01232 expression. **c** LINC01232 expression was detected in 40 pairs of PAAD tissues and adjacent normal tissues. **d** qRT-PCR assay was implemented to estimate the expression level of LINC01232 in normal cell line (HPDE6-C7) and PAAD cells (CAPAN-1, BxPC-3, JF305, PANC-1, and SW1990). *P < 0.05, **P *<* 0.01

According to the analysis results of TCGA database, LINC01232 expression in was higher in PAAD tissues than that in normal tissues (Fig. 1a). Furthermore, the correlation between LINC01232 level and the prognosis of TCGA PAAD patients was also identified. Survival analysis revealed that PAAD patients with high level of LINC01232 exhibited lower overall survival than those with low LINC01232 level (Fig. 1b). To validate the results of TCGA database, LINC01232 expression was subsequently detected in 40 pairs of tissues obtained from PAAD patients. Similarly, LINC01232 presented higher expression level in PAAD samples compared to that in adjacent normal samples (Fig. 1c). Consistently, the expression level of LINC01232 in PAAD cell lines was found to be higher compared to the normal cell line (Fig. 1d).

### Silencing of LINC01232 hampers cell proliferation and promoted cell apoptosis in PAAD

**Fig. 2:**
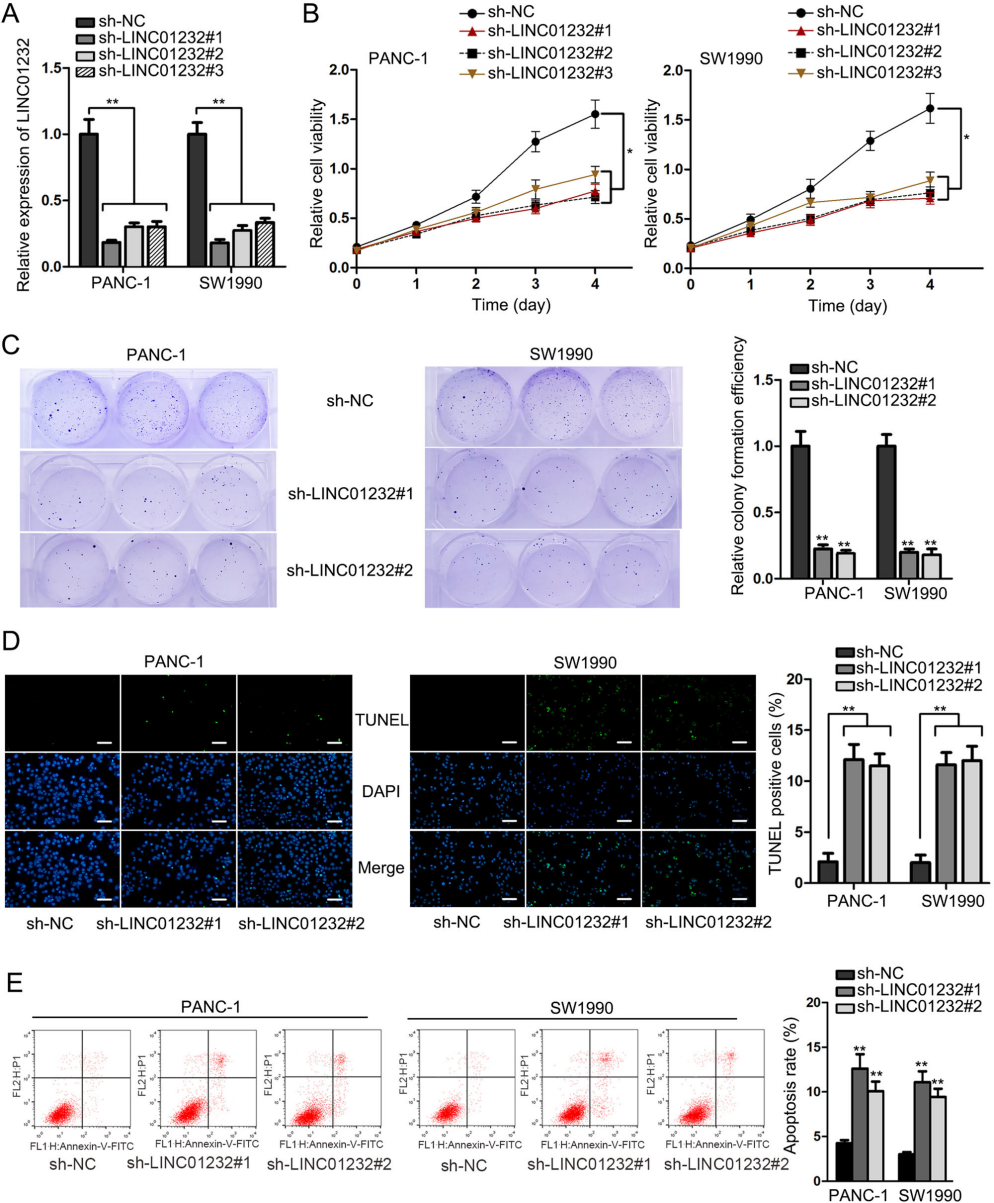
Silencing of LINC01232 hampered cell proliferation and promoted cell apoptosis in PAAD. **a** The efficiency of LINC01232 knockdown in PANC-1 and SW1990 cells was verified by qRT-PCR. **b**, **c** Cell proliferative ability was assessed by MTT and colony formation assays after LINC01232 knockdown. **d**, **e** TUNEL-staining assay and flow cytometry analysis were adopted to determine the apoptosis of PANC-1 and SW1990 cells after indicated transfection. *P < 0.05, **P *<* 0.01

In order to unveil the specific role of LINC01232, LINC01232 was knocked down in PANC-1 and SW1990 cells by transfection with sh-LINC01232#1/2/3 plasmids (Fig. 2a). MTT and colony formation assays demonstrated that inhibition of LINC01232 contributed to the overt diminution of cell proliferation (Fig. 2b, c). In addition, TUNEL-staining assay manifested that the proportion of TUNEL-positive cells was increased due to depletion of LINC01232 (Fig. 2d). Further, increased apoptosis rate was further detected in LINC01232-downregulated cells by using flow cytometry analysis (Fig. 2e). Collectively, we concluded that LINC01232 acted as an oncogene in PAAD by inducing cell proliferation and suppressing cell apoptosis.

### Knockdown LINC01232 led to the repression of cell migration, invasion, and EMT in PAAD

**Fig. 3:**
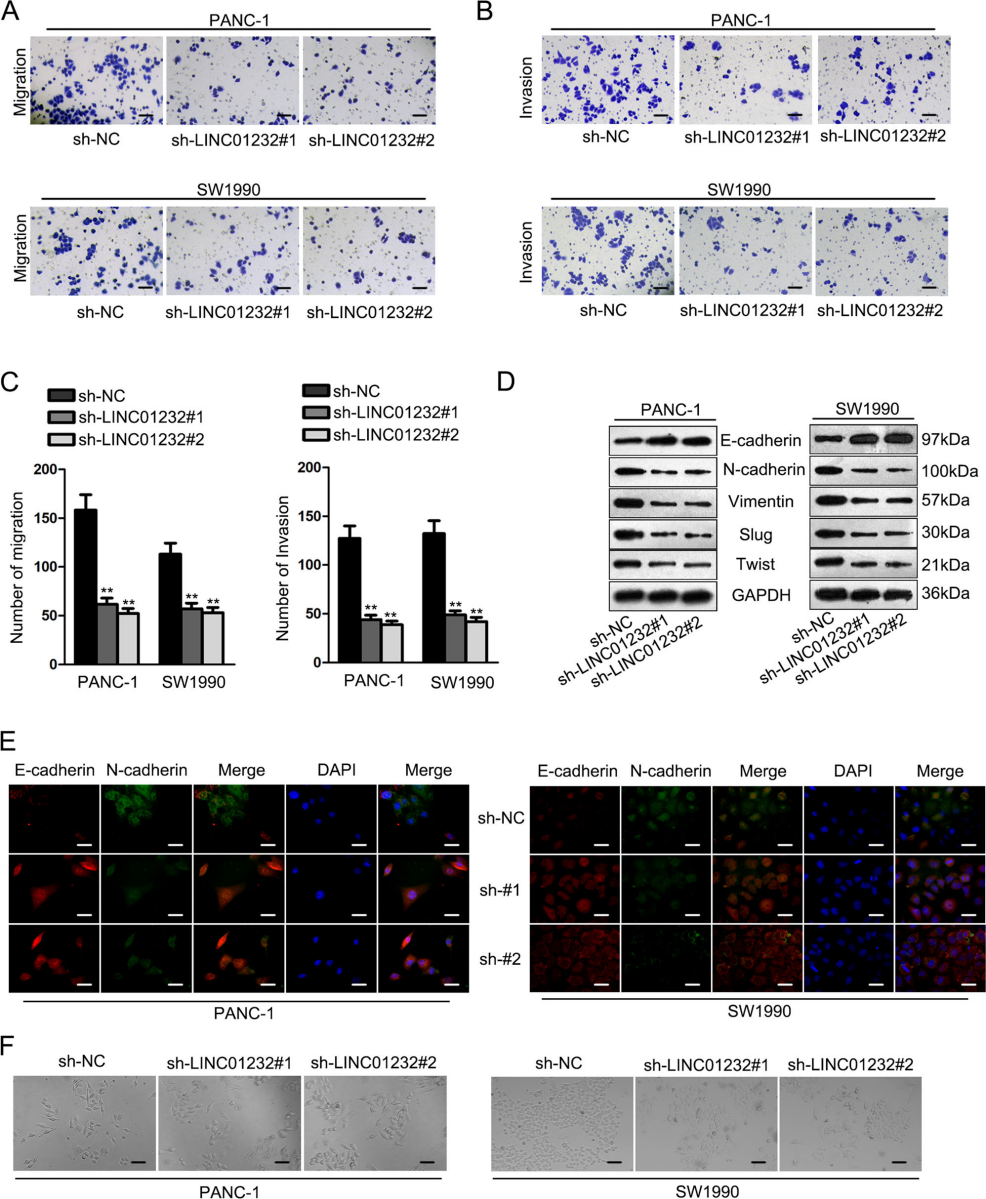
Knockdown LINC01232 led to the repression of cell migration, invasion and EMT in PAAD. **a**–**c** Transwell assays were carried out to detect cell migration and invasion in LINC01232-downregulated PAAD cells. **d** The levels of EMT-related proteins was measured by western blotting. **e** Immunofluorescence assay was applied to further confirm the levels of E-cadherin and N-cadherin in PANC-1 and SW1990 cells. **f** The phenotype of PANC-1 and SW1990 cells transfected with different plasmids was observed. **P *<* 0.01

To further probe the effects of LINC01232 on cell migration and invasion, we carried out transwell assays and verified that the migratory and invasive capacities of PANC-1 and SW1990 cells were restrained on account of LINC01232 downregulation (Fig. 3a–c). EMT has been proven to be of paramount significance in cell processes of migration and invasion^16^. Here, western blot was adopted to evaluate the protein level of EMT markers. Our results illuminated that epithelial marker, E-cadherin, was increased in PANC-1 and SW1990 cells with LINC01232 knockdown, while the levels of mesenchymal markers N-cadherin, Vimentin and related transcription factors Snail and Twist were prominently decreased by depletion of LINC01232 (Fig. 3d). In agreement with these findings, immunofluorescent assays certified that LINC01232 silencing resulted in enhanced E-cadherin level and the reduced expression of N-cadherin in both PANC-1 and SW1990 cells (Fig. 3e). In addition, we observed the phenotype of PANC-1 and SW1990 cells and found that knockdown of LINC01232 inducing EMT phenotype into MET phenotype (Fig. 3f). By the large, we expounded that knockdown of LINC01232 retarded the migration and invasion of pancreatic cancer cells via reversing EMT processes.

### LINC01232 positively regulated the expression of TM9SF2

**Fig. 4:**
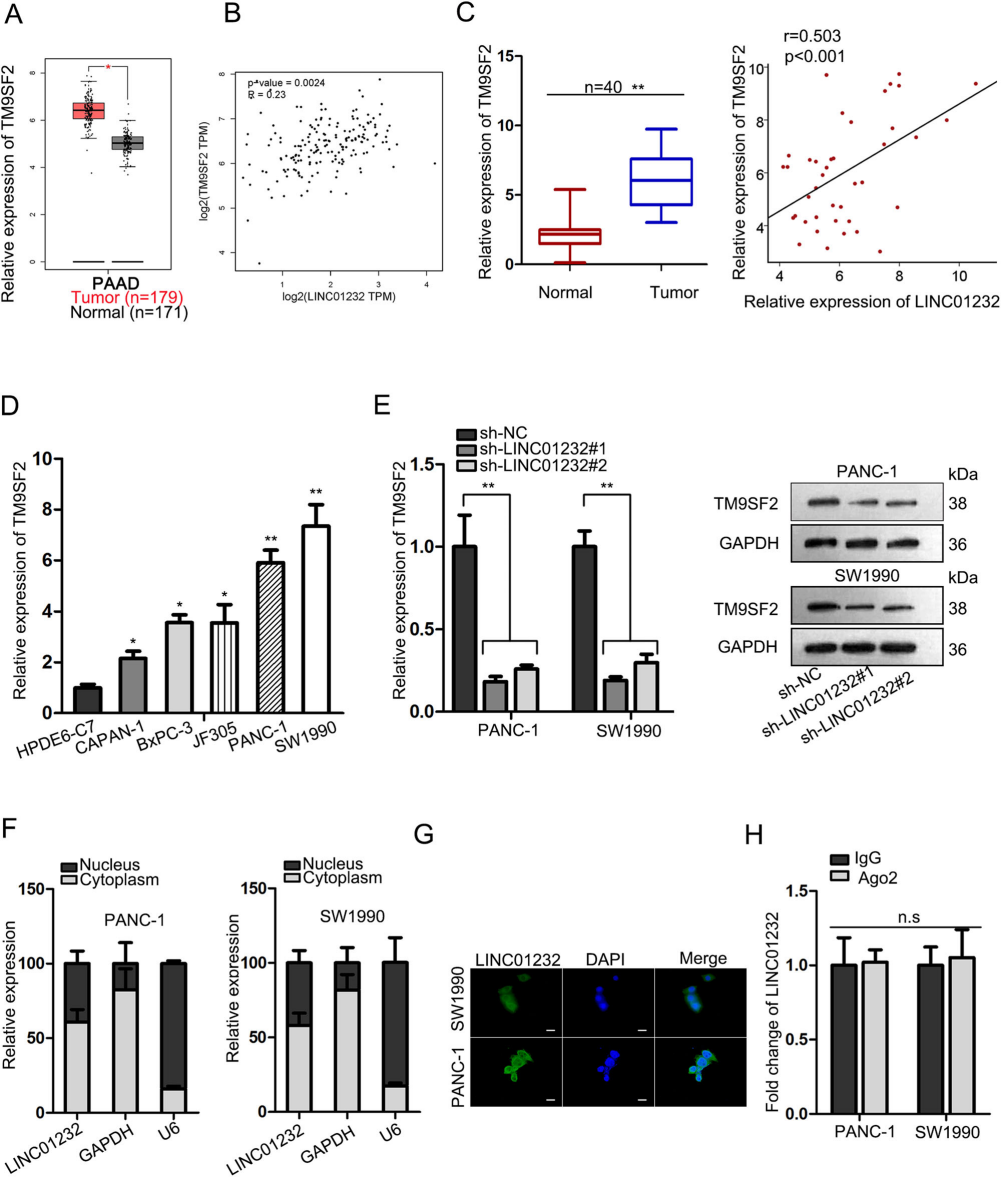
LINC01232 positively regulated the expression of TM9SF2. **a** Upregulation of TM9SF2 expression in TCGA PAAD tissues. **b** TCGA correlation analysis of LINC01232 and TM9SF2. **c** TM9SF2 expression and its correlation with LINC01232 in 40 PAAD tissues. **d** TM9SF2 expression in normal cell line and PAAD cell lines. **e** mRNA and protein level of TM9SF2 were examined in PAAD cells transfected with LINC01232-specific shRNA and control shRNA. **f**, **g** Subcellular fractionation and FISH assay were carried out to determine the nuclear-cytoplasmic fractionation of LINC01232 in PAAD cells. **h** Ago2-RIP experiment was utilized to estimate the capability of LINC01232 to combine with RISC complex. *P < 0.05, **P *<* 0.01

By browsing the UCSC website, we found that TM9SF2 was a gene near LINC01232 (Fig. S1A). Based on TCGA data, the upregulation of TM9SF2 expression was observed in pancreatic cancer tissues (Fig. 4a). Moreover, it was showed by TCGA correlation analysis that there was a positive expression association between TM9SF2 and LINC01232 in PAAD samples (Fig. 4b). Moreover, we also observed that TM9SF2 was expressed at a higher level in 40 PAAD samples compared to adjacent normal controls, which was consistent with LINC01232 (Fig. 4c). The qRT-PCR assay delineated that the expression of TM9SF2 in PAAD cells was higher than that in normal cells (Fig. 4d). Therefore, we intended to examine the potential regulatory effect of LINC01232 on TM9SF2 expression. Accordingly, we detected the mRNA and protein level of TM9SF2 in cells transfected with LINC01232-specific shRNA and control shRNA. Both levels were decreased by the knockdown of LINC01232 (Fig. 4e). In addition, subcellular fractionation analysis indicated that LINC01232 was predominantly expressed in cytoplasm (Fig. 4f). And this result was further validated by FISH assay (Fig. 4g). In turn, we surmised that LINC01232 functioned as a ceRNA to regulate TM9SF2. However, RIP experiments illustrated that LINC01232 had no ability to bind with Ago2, suggesting that LINC01232 was unable to combine with RNA-induced silencing complex (RISC) (Fig. 4h). To determine the role of TM9SF2 in cellular processes, we silenced it in PANC-1 and SW1990 cells (Fig. S1B). After loss-of function analysis, we determined that silencing of TM9SF2 efficiently suppressed cell proliferation (Fig. S1C, D). Furthermore, migratory ability of PAAD cells was weakened after TM9SF2 was knocked down (Fig. S1E). EMT markers were also tested in indicated cells. As a result, we observed that EMT process was reversed by the knockdown of TM9SF2 (Fig. S1F). On the whole, we concluded that TM9SF2 was positively regulated by LINC01232 and promoted PAAD cell proliferation and migration.

### LINC01232 recruited EIF4A3 to stabilize TM9SF2

**Fig. 5:**
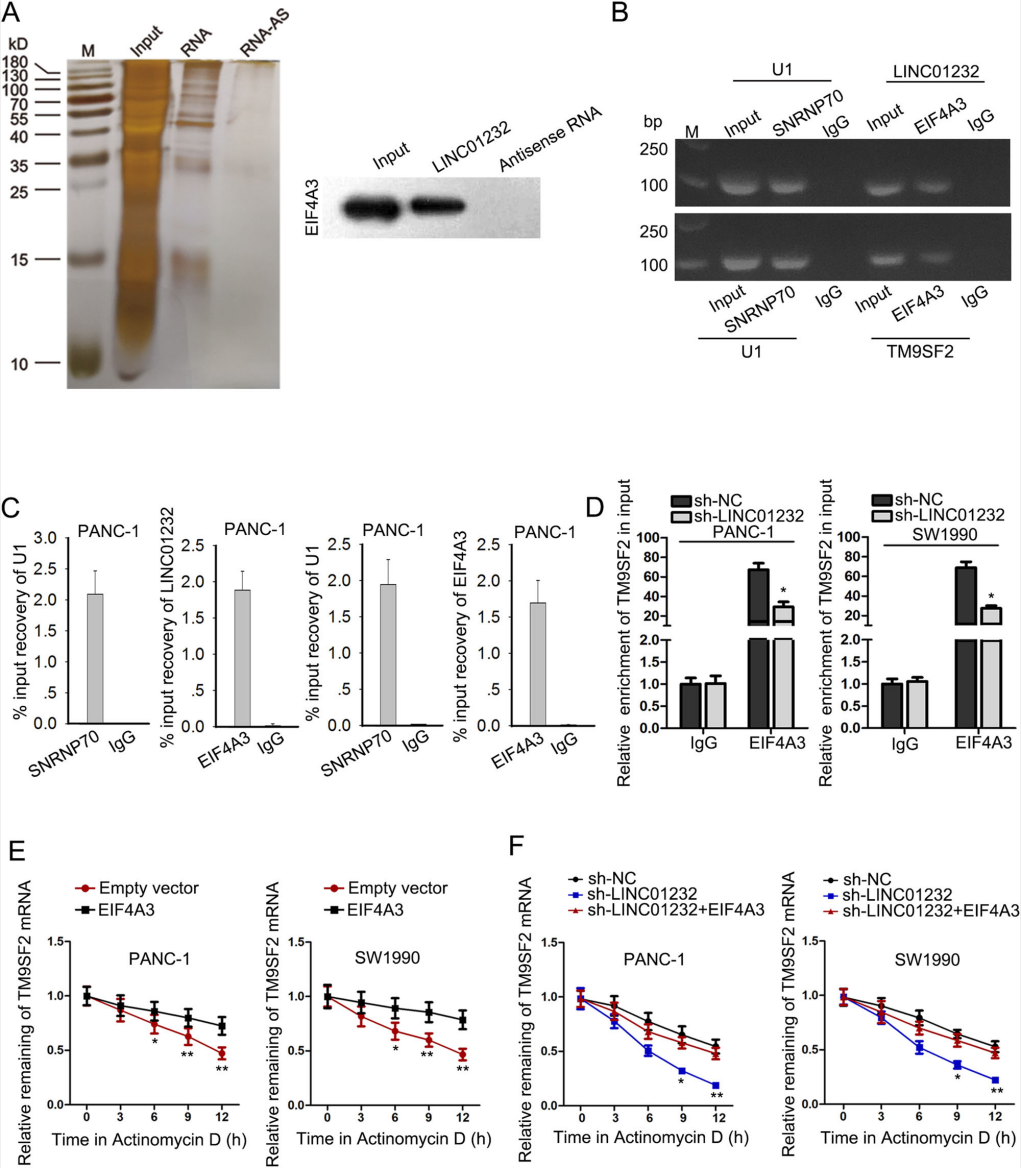
LINC01232 recruited EIF4A3 to stabilize TM9SF2. **a** Pull-down silver staining and the peak map for mass spectrometry analysis were used to demonstrate the interaction between EIF4A3 and LINC01232. **b**, **c** RIP assay was applied to validate the binding of EIF4A3 to LINC01232 and TM9SF2. **d** The effect of LINC01232 knockdown on the binding of EIF4A3 to TM9SF2 was assessed by RIP assay. **e**, **f** After Actinomycin D treatment, the mRNA stability of TM9SF2 in indicated cells was determined by qRT-PCR. *P < 0.05

To obtain a more particular knowledge of whether LINC01232 modulated TM9SF2, we conducted bioinformatics analysis. Pull-down silver staining and mass spectrometry analysis showed that there are some RBPs that can bind to LINC01232 (Fig. 5a). The interaction between EIF4A3 and LINC01232 was further validated and shown by using western blot analysis. The peak map showed the enrichment of EIF4A3 in biotin-labeled-LINC01232 (Fig. S2A). To determine whether LINC01232 had regulatory effect on EIF4A3 expression, we examined both mRNA and protein levels of EIF4A3 in LINC01232-downregulated PAAD cells. No significant expression change was observed (Fig. S2B), indicating that LINC01232 potentially recruited EIF4A3 to regulate their downstream mRNAs. In addition, the expression of EIF4A3 was remarkably increased in TAGA and obtained PAAD tissues and positively correlated with TM9SF2 (Fig. S2C, D). Similarly, we detected relative high-expression level of EIF4A3 in PAAD cell lines (Fig. S1E). Then, we silenced EIF4A3 expression and observed that the level of TM9SF2 was dropped by EIF4A3 depletion (Fig. S1E, F). Thus, we chose EIF4A3 for further analysis. Furtherly, RIP assay validated that EIF4A3 could interact with both LINC01232 and TM9SF2 (Fig. 5b, c). More importantly, the enrichment of TM9SF2 in anti-EIF4A3 precipitates was diminished owing to LINC01232 silencing (Fig. 5d). It is known that interaction between lncRNAs and RBPs can induce the change of mRNA stability. Thus, we tested the mRNA stability of TM9SF2 in cells treated with Actinomycin D after transfections. As expected, overexpression of EIF4A3 led to the fortified mRNA stability of TM9SF2 (Fig. 5e). And LINC01232 knockdown decreased TM9SF2 mRNA stability and the rebound of TM9SF2 stability occurred with upregulation of EIF4A3 (Fig. 5f). Taken together, these findings revealed that LINC01232 modulated TM9SF2 expression through recruiting EIF4A3 to enhance the mRNA stability of TM9SF2.

### LINC01232 and TM9SF2 synergistically promoted PAAD progression

**Fig. 6:**
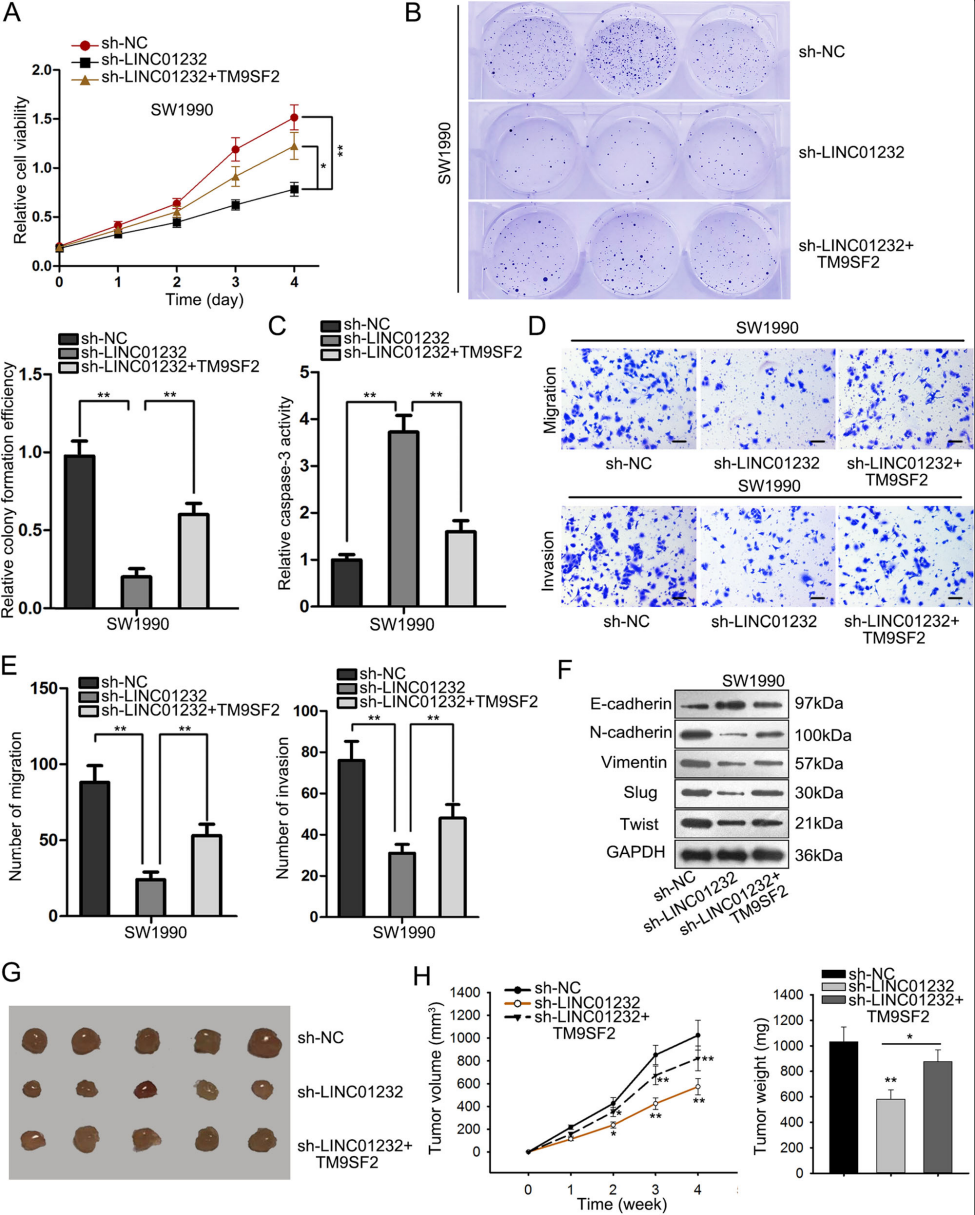
LINC01232 and TM9SF2 synergistically promoted PAAD progression. **a**, **b** Cell proliferation in cells transfected with sh-NC, sh-LINC01232, or co-transfected with sh-LINC01232 and TM9SF2 expression vector was measured by the MTT and colony formation assays. **c** Cell apoptosis rate was evaluated by caspase-3 activity assay. **d**, **e** Cell migratory and invasive capacities were determined by transwell assays. **f** EMT process was estimated by western blotting. **g** Tumors derived from cells transfected with different plasmids. **h**, **i** Tumor volume and tumor weight in different groups were calculated and shown. *P < 0.05, **P *<* 0.01

Rescue experiments were performed to explore whether LINC01232 affected the tumorigenesis and development of pancreatic cancer by TM9SF2. As displayed in Fig. 6a, b, cell proliferation repressed by silencing of LINC01232 was prompted when TM9SF2 was overexpressed. It was stated that ectopic expression of TM9SF2 abolished the promotion of cell apoptosis caused by LINC01232 knockdown (Fig. 6c). Furthermore, TM9SF2 upregulation boosted cell migration and invasion inhibited by LINC01232 attenuation (Fig. 6d, e). In concert with mentioned results, overexpression of TM9SF2 reversed the influence of LINC01232 depletion on the expression of EMT-relevant proteins (Fig. 6f). Through in vivo experiments, we further determined that tumor growth in sh-LINC01232 group was slower than sh-NC group. Whereas, tumor growth was faster after co-transfection with TM9SF2 expression vector (Fig. 6g, h). To sum up, LINC01232 executed carcinogenic properties in pancreatic cancer via regulation of TM9SF2.

### SP1 acted as a transcriptional activator of LINC01232 and TM9SF2 in PAAD cells

**Fig. 7:**
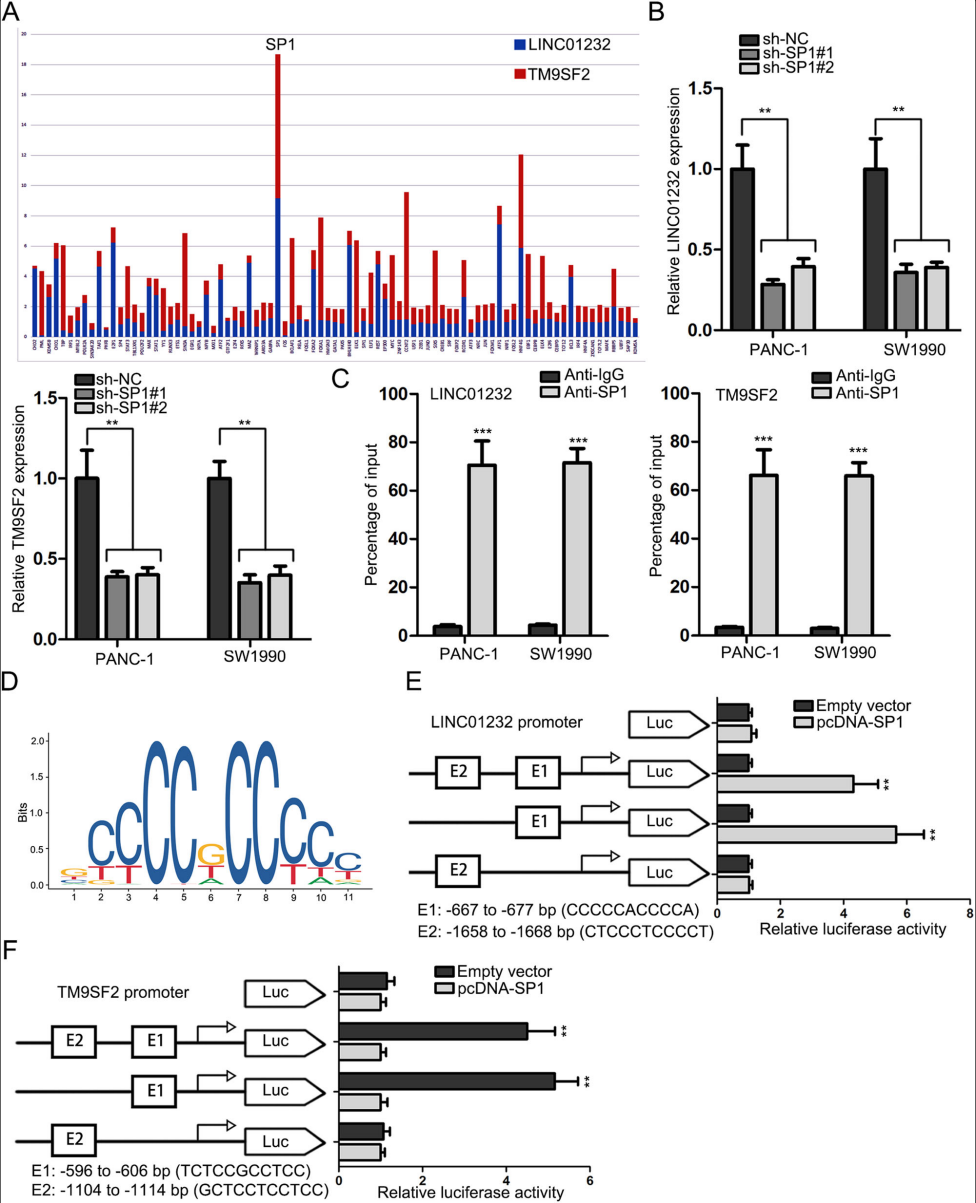
SP1 acted as a transcriptional activator of LINC01232 and TM9SF2 in PAAD cells. **a** After transcription factors were respectively overexpressed, we observed that upregulation of SP1 led to the most significant increase of TM9SF2 and LINC01232 expression. **b** The levels of TM9SF2 and LINC01232 in response to inhibition of SP1 were detected by qRT-PCR. **c** ChIP assay revealed the binding capability of SP1 to LINC01232 and TM9SF2 promoters. **d** DNA motif of SP1. **e**, **f** Specific binding sites of SP1 for LINC01232 and TM9SF2 promoters were validated by luciferase reporter gene assays. *P < 0.05, **P *<* 0.01, ***P *<* 0.001

Through employment of UCSC database, we screened out 85 candidate transcription regulators for both TM9SF2 and LINC01232. To identify the co-regulators, we overexpressed all these candidate genes to detect the expression change of both TM9SF2 and LINC01232. As presented in Fig. 7a, SP1 overexpression led to the most significant upregulation of TM9SF2 and LINC01232. Importantly, SP1 was upregulated in TCGA PAAD samples and positively correlated with LINC01232 and TM9SF2 (Fig. S3A, B). High level of SP1 indicated poor overall survival of PAAD patients (Fig. S3C). Upregulation of SP1 was further determined in 40 PAAD tissues (Fig. S3D). The expression correlation between SP1 and LINC01232 or TM9SF2 was also analyzed in PAAD samples and was found to be positive (Fig. S3E). To make further confirmation, SP1 expression was silenced in two PAAD cell lines and transfection efficiency was verified by qRT-PCR (Fig. S3F). Moreover, SP1 was found to be unable to regulate mRNA stability of TM9SF2 (Fig. S3G). It was proofed that depletion of SP1 decreased the levels of LINC01232 and TM9SF2 (Fig. 7b). Then, we determined the affinity of SP1 to the promoter of LINC01232 or TM9SF2 by ChIP assay. It was corroborated by ChIP experiments that SP1 directly bound with LINC01232 and TM9SF2 promoters (Fig. 7c). To further identify their interactions, we obtained the DNA motif of SP1 and the putative binding sites of SP1 to LINC01232 and TM9SF2 promoters from JASPAR database (Fig. 7d). To determine the detailed binding sites, luciferase reporter gene assays were carried out. Results manifested that the region between −667 and −677 bp (E1) on the LINC01232 promoter was responsible for SP1-mediated transcriptional activation (Fig. 7e). Whereas, sequence from −596 to −606 bp of TM9SF2 promoter was the key binding site for SP1 (Fig. 7f). In a word, the transcriptional activation of LINC01232 and TM9SF2 was mediated by SP1.

## Discussion

Pancreatic cancer is one of the most lethal diseases in digestive system and ranks as the fourth leading reason of cancer death^17^. Pancreatic cancer is characterized by lack of early diagnosis and poor prognosis and its morbidity is increasing annually worldwide^18,19^. Although constant innovation of therapeutic options has been achieved, 5-year survival rate of pancreatic cancer patients remains less than 5%^20^. Hence, it is of great significance to identify molecular targets associated with the development of PAAD.

Mounting evidence has revealed that lncRNAs participate in biological processes of diverse cancers by chromatin modification and genomic imprinting, transcriptional activation, regulation at post-transcriptional level^21^. For instance, long noncoding RNA DANCR promotes the progression of non-small-cell lung cancer by inhibiting p21 expression^22^. LncRNA MALAT1 binds chromatin remodeling subunit BRG1 to epigenetically promote inflammation-related hepatocellular carcinoma progression^23^. Silencing of DLEU2 suppresses pancreatic cancer cell proliferation and invasion by upregulating miR-455^24^. Searching from TCGA database, we determined that LINC01232 is dysregulated in PAAD samples and had potential to regulate prognosis. Previously, LINC01232 has been reported to be highly expressed in oral carcinoma tissues and associated with the shorter survival rate^25^. However, to our knowledge, the potential of LINC01232 in PAAD progression remains to be further investigated. Our findings testified that high expression of LINC01232 in PAAD cells. Knockdown of LINC01232 hampered cell proliferation, migration, invasion, and EMT whereas boosted cell apoptosis. Thus, we speculated that LINC01232 exerted oncogenic property in PAAD progression.

TM9SF2 is an evolutionarily conserved transmembrane protein and has been identified as a glycolipid-regulating factor and a novel colorectal cancer oncogene^14,26^. However, the function of TM9SF2 in tumorigenesis and progression of PAAD still needs to be delineated. After bioinformatics analysis, we knew that TM9SF2 is the nearby gene of LINC01232. Intriguingly, TM9SF2 was also upregulated in TCGA PAAD samples and positively correlated with LINC01232. Then, we validated that LINC01232 positively regulated the expression of TM9SF2. Mechanistically, lncRNAs can modulate target gene expression via miRNA response elements^27,28^. Although LINC01232 was predominantly expressed in cytoplasm, it cannot bind with RISC complex. Thus, LINC01232 regulated TM9SF2 potentially via other mechanisms.

Based on previous reports, lncRNAs can regulate the mRNA stability of their downstream genes by recruiting RBPs^29–32^. In current study, pull-down assay and mass-spectrometry analysis detected the RBPs that could interact with LINC01232. Among which, eukaryotic translation initiation factor 4A3 (EIF4A3), a key exon junction complex component, can cause mRNA decay and regulate protein expression at the translational and post-translational levels^33,34^. Moreover, several researches have revealed that EIF4A3 executes carcinogenic properties in glioblastoma and ovarian cancer^35,36^. TCGA data presented that upregulated EIF4A3 positively correlated with LINC01232 and TM9SF2 in PAAD samples. Further mechanism investigation revealed that binding of EIF4A3 to TM9SF2 was affected by the expression level of LINC01232. After detection, we determined that the mRNA stability of TM9SF2 was positively regulated by LINC01232/EIF4A3 axis. Hereto, we elucidated that LINC01232 recruited EIF4A3 to stabilize TM9SF2 mRNA. Furthermore, TM9SF2 involved in LINC01232-mediated PAAD progression.

**Fig. 8:**
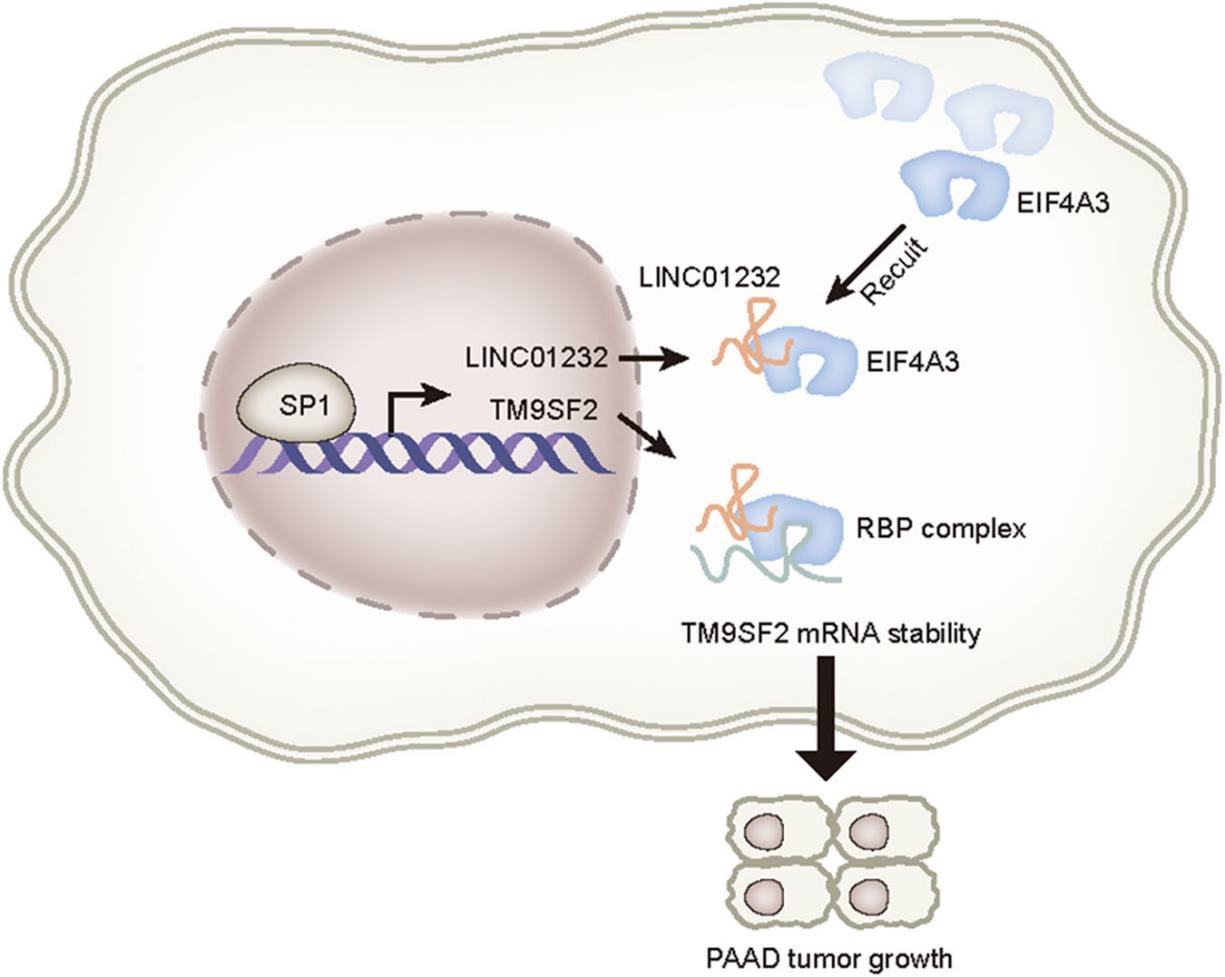
Graphical abstract illustrated the function and molecular mechanism of LINC01232 in PAAD progression

Since upregulation of LINC01232 and TM9SF2 promoted PAAD progression, we further analyzed whether there are some molecular mechanisms led to the upregulation of LINC01232 and TM9SF2. It is known that transcriptional activation is of great significance for dysregulation of genes. In our present study, we determined that SP1 is a transcription activator for both LINC01232 and TM9SF2. Upregulation of SP1 in PAAD cells induced the overexpression of LINC01232 and TM9SF2. More importantly, SP1 was upregulated in TCGA PAAD samples and correlated with poor patients’ prognosis. To summarize, this study was the first to unveil the role and molecular mechanism of LINC01232, expounding that SP1-induced LINC01232 exerted oncogenic activities in PAAD via recruiting EIF4A3 to upregulate TM9SF2 expression (Fig. 8). All these findings may provide a promising molecular target for PAAD treatment.

## Supplementary information

Figure S1

Figure S2

Figure S3

Supplementary figure legends.
