# Supplementary figures and images for: LINC01232 exerts oncogenic activities in pancreatic adenocarcinoma via regulation of TM9SF2
Source: Cell Death Dis. 2019 Sep 20;10(10):698. doi: 10.1038/s41419-019-1896-3 (PMC6754375; doi:10.1038/s41419-019-1896-3)

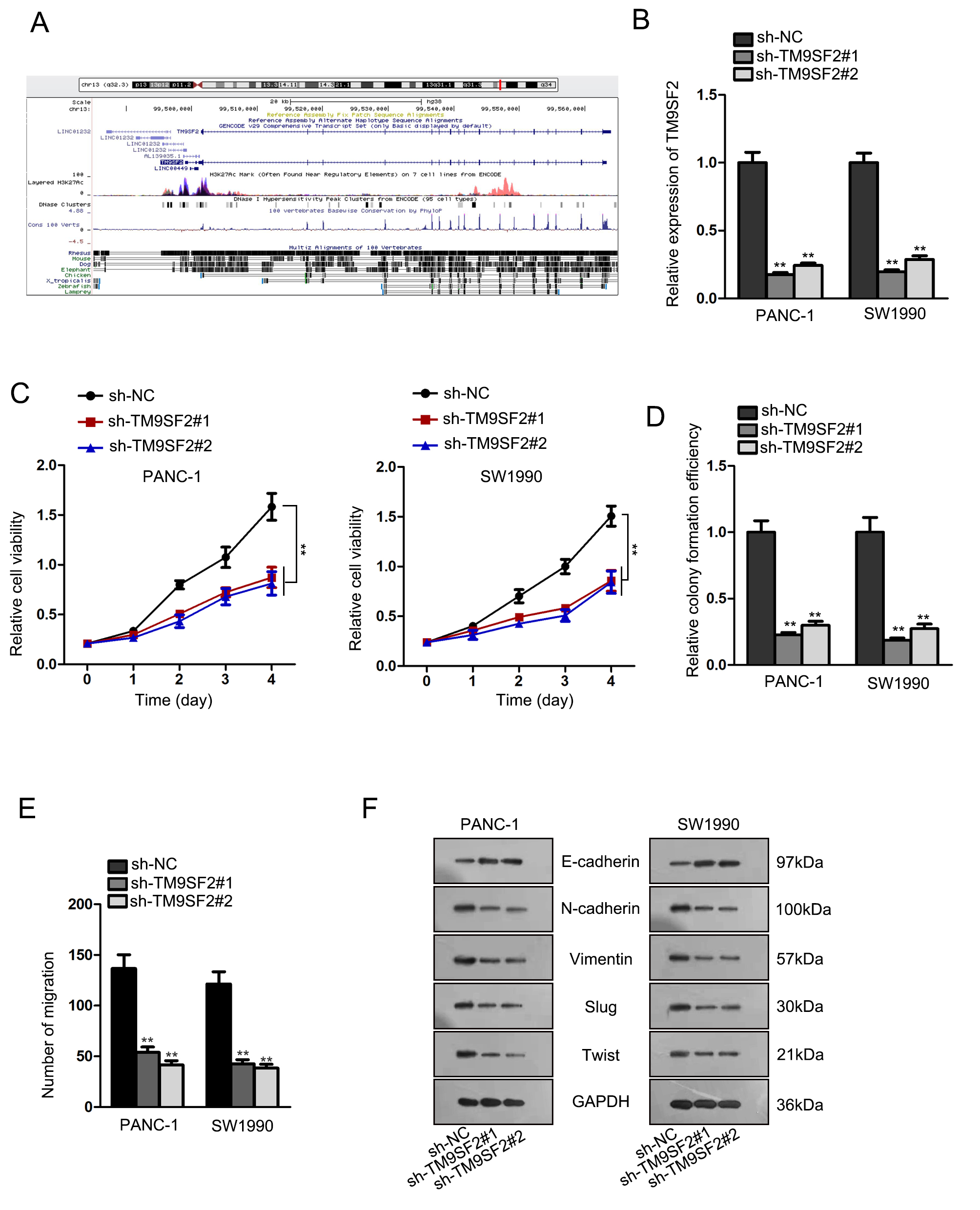

Supplement: Supplementary file 1 — Figure S1 [file 41419_2019_1896_MOESM1_ESM.tif]

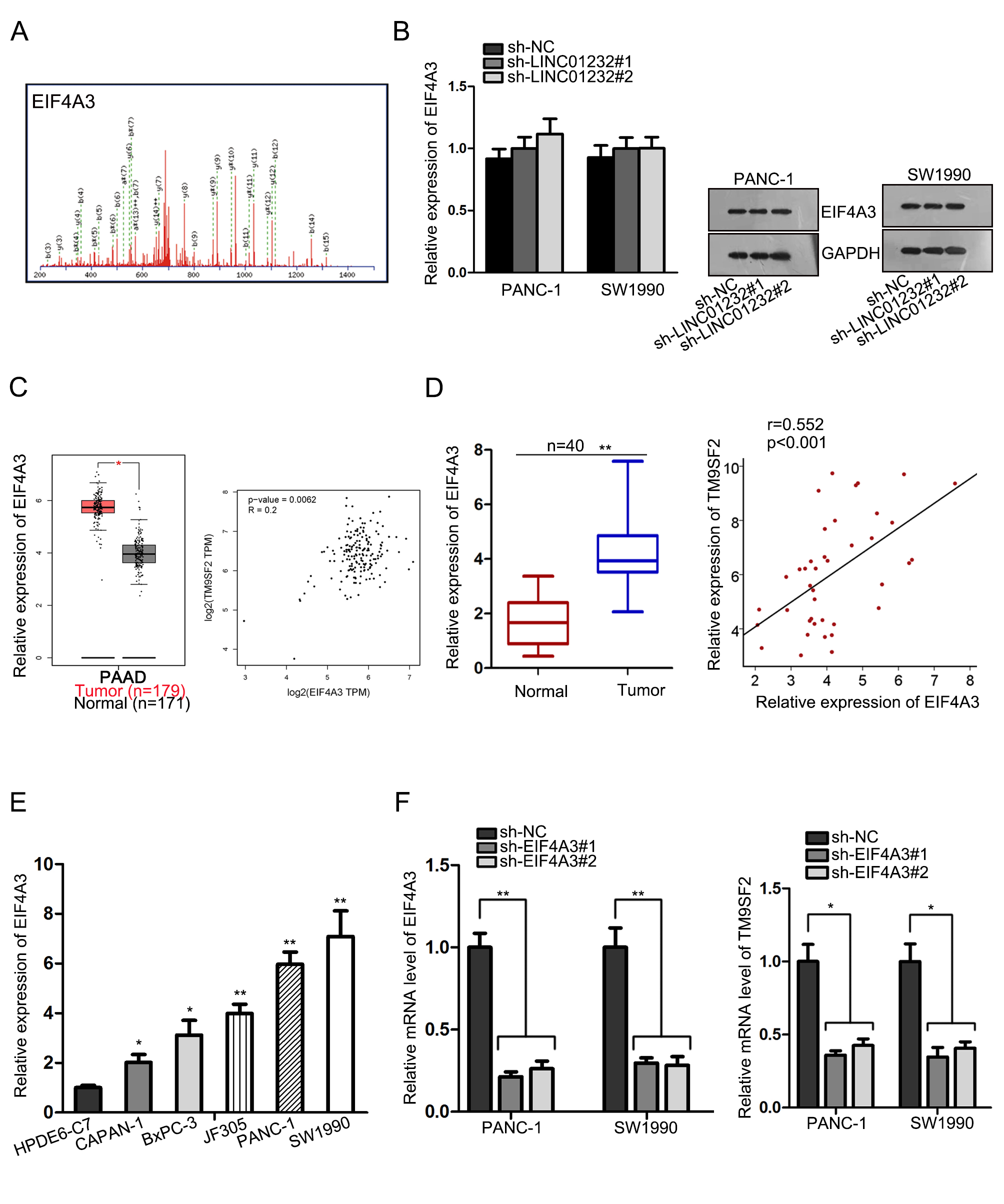

Supplement: Supplementary file 2 — Figure S2 [file 41419_2019_1896_MOESM2_ESM.tif]

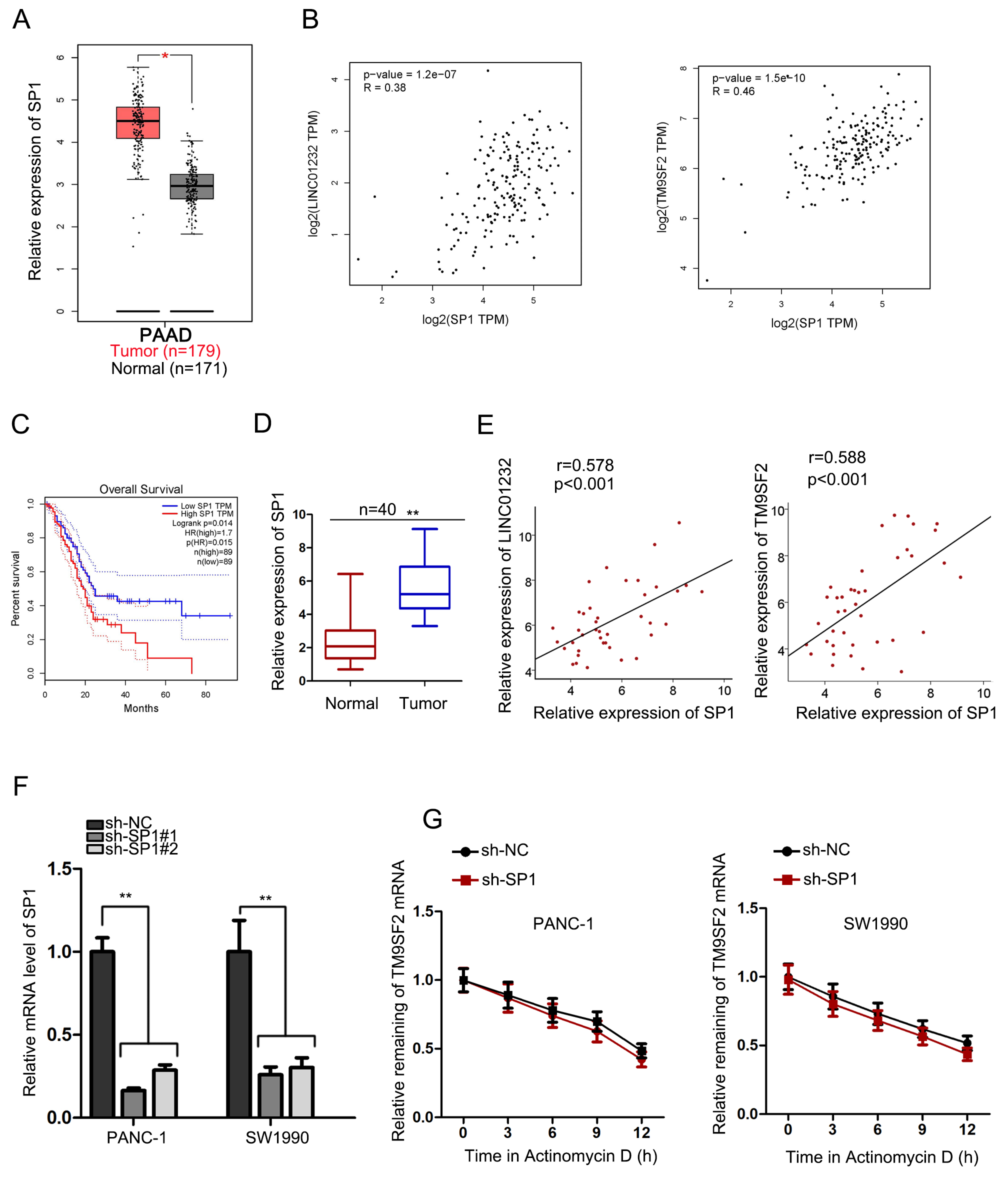

Supplement: Supplementary file 3 — Figure S3 [file 41419_2019_1896_MOESM3_ESM.tif]
