# Supplementary material for: LINC01232 exerts oncogenic activities in pancreatic adenocarcinoma via regulation of TM9SF2
Source: Cell Death Dis. 2019 Sep 20;10(10):698. doi: 10.1038/s41419-019-1896-3 (PMC6754375; doi:10.1038/s41419-019-1896-3)
Supplement: Supplementary file 4 — Supplementary figure legends. [file 41419_2019_1896_MOESM4_ESM.docx]

**Supplementary figure legends**

**Figure S1** (A) The location of LINC01232 and TM9SF2 was identified by browsing the UCSC website. (B) Transfection efficiency was determined after cells were transfected with TM9SF2-specific shRNAs or control shRNA. (C-D) MTT and colony formation assays were carried out to detect proliferative ability of PAAD cells after silencing of TM9SF2. (E) Transwell migration assay was applied to measure migratory ability of TM9SF2-downregulated PAAD cells. (F) EMT markers were also tested in indicated cells by western blot assay. ^*^*P* < 0.05, ^**^*P* < 0.01.

**Figure S2** (A) The peak map showed the enrichment of EIF4A3 in biotin-labeled-LINC01232. (B) Both mRNA and protein levels of EIF4A3 were detected in LINC01232-downregulated PAAD cells. (C) TCGA data presented that the expression of EIF4A3 in PAAD tissues and its correlation with TM9SF2. (D) EIF4A3 expression and its correlation with TM9SF2 in 40 PAAD tissues. (E) qRT-PCR analysis was conducted to examine EIF4A3 expression in normal cell and PAAD cells. (F) Knockdown efficiency of EIF4A3 in PAAD cells and expression change of TM9SF2 were verified by qRT-PCR. ^*^*P* < 0.05, ^**^*P* < 0.01.

**Figure S3** (A) TCGA data exposed SP1 expression in PAAD samples. (B) Positive expression association between SP1 and LINC01232 or TM9SF2. (C) TCGA data that poor prognosis of pancreatic cancer patients correlated with high expression of SP1. (D) Upregulation of SP1 was determined in 40 PAAD tissues. (E) The expression correlation between SP1 and LINC01232 or TM9SF2 was analyzed in PAAD samples by Pearson correlation analysis. (F) Transfection efficiency for control shRNA and SP1-specific shRNAs in PAAD cells. (G) mRNA stability of TM9SF2 was measured in cells transfected with sh-NC or sh-SP1. ^*^*P* < 0.05, ^**^*P* < 0.01.
